# The deubiquitinase ElaD is present in the majority of *Escherichia coli* strains

**DOI:** 10.3389/fmicb.2025.1681308

**Published:** 2025-10-17

**Authors:** Xinyu Wang, Weiqi Guo, Jiangang Hu, Beibei Zhang, Jingjing Qi, Mingxing Tian, Yanqing Bao, Lei Deng, Shaohui Wang

**Affiliations:** Shanghai Veterinary Research Institute, Chinese Academy of Agricultural Sciences (CAAS), Shanghai, China

**Keywords:** *Escherichia coli*, deubiquitinase, ElaD, distribution, pathotype

## Abstract

**Background:**

Pathogens employ a variety of effectors to modulate key host signaling pathways, thereby facilitating bacterial survival and enhancing pathogenicity. Despite lacking a complete ubiquitin system of their own, bacterial effectors frequently function as ubiquitin ligases or deubiquitinases (DUBs) to disrupt the eukaryotic ubiquitin machinery. DUBs have been found in a variety of bacteria, including ElaD, which has recently been recognized as a DUB in *Escherichia coli* (*E. coli*). However, the distribution and evolutionary analyses of ElaD in different *E. coli* remains largely unknown.

**Methods:**

We retrieved and analyzed the *elaD* gene sequences of 530 *E. coli* strains. Then, molecular characterization of each strain was determined. According to all the statistical information, the distribution of *elaD* gene in *E. coli* was comprehensively investigated, and the relationship between *elaD* and *E. coli* pathotypes, serotypes, phylogenetic groups and MLSTs was analyzed. Phylogenetic tree was also constructed to analyze the evolutionary relationships between different ElaD.

**Results:**

Our findings demonstrate that the *elaD* gene was present in 66.60% (353/530) of both pathogenic and nonpathogenic *E. coli* strains. *elaD* gene is predominantly found in the O157, O26, O139 and O8 serotypes. The majority of *elaD*-positive strains belonged to phylogenetic groups B1, A, E and D, with the predominant sequence types being ST11, ST21, ST10, ST1 and ST69. ElaD from different strains clustered in the phylogenetic tree in a correlation with O serotypes and phylogenetic groups. In addition, ElaD of some branches showed premature translation termination.

**Conclusion:**

The widespread occurrence of the *elaD* gene among various *E. coli* strains suggests its potential significance in *E. coli*, although its precise functional role remains to be elucidated.

## Introduction

Pathogenic *Escherichia coli* (*E. coli*) capable of causing severe diseases from gastroenteritis to extraintestinal infections according to the acquisition of a mixture of comprehensive mobile genetic elements, which encode virulence factors (Frost et al., 2005; Pakbin et al., 2021). According to the different pathogenesis and lesion location, it can be divided into intestinal pathogenic *E. coli* (IPEC) and extraintestinal pathogenic *E. coli* (ExPEC) (Russo and Johnson, 2003). Among them, IPEC includes enteropathogenic *E. coli* (EPEC), Shiga toxin-producing *E. coli* (STEC), enterohaemorrhagic *E. coli* (EHEC), enterotoxigenic *E. coli* (ETEC), enteroinvasive *E. coli* (EIEC), enteroaggregative *E. coli* (EAEC), diffusely adherent *E. coli* (DAEC), as well as a new pathotype, adherent invasive *E. coli* (AIEC), which mainly causes diarrhea and intestinal diseases; newborn meningitis *E. coli* (NMEC), uropathogenic *E. coli* (UPEC), avian pathogenic *E. coli* (APEC), which can cause human urinary tract infections, neonatal meningitis, avian respiratory tract or systemic infections, are considered ExPEC (Sora et al., 2021). *E. coli* are also characterized by the serotype, phylogenetic group and MLST (multilocus sequence typing) to which they belong (Riley, 2020). Some pathogenic *E. coli* infecting humans can cause serious morbidity and mortality worldwide (Clements et al., 2012). Animal pathogenic *E. coli* (such as APEC), in addition to bringing huge economic losses to farms, may also be transmitted to humans through feces, drinking water or undercooked meat, posing a potential health threat, which has a tremendous burden on public health (Hu et al., 2022; Tapader et al., 2019).

The binding of ubiquitin molecules to eukaryotic proteins helps to regulate post-translational modifications, thus influencing and participating in the majority of cellular processes such as cell cycle, apoptosis and cell signal transduction (Kerscher et al., 2006; Song and Luo, 2019). Ubiquitination is a strictly regulated and reversible process (Liu et al., 2005). On the one hand, ubiquitin-activating enzyme, ubiquitin-conjugating enzyme and ubiquitin ligase catalyze a sophisticated three-step enzymatic cascade to add one ubiquitin (Ub) or a chain of Ubs to the substrate, which helps to complete the process of post-translational modification of proteins; on the other hand, deubiquitinases (DUBs) can remove Ub from the substrate proteins (Di Gregorio et al., 2023; Fang et al., 2023; Wolberger, 2014). DUBs are involved in counterbalancing and proofreading ubiquitin processes, as well as recycling ubiquitins (Snyder and Silva, 2021). The dynamic balance between DUBs and ubiquitin ligases is essential for many aspects of cellular processes (Komander et al., 2009; Li et al., 2023).

Increasing studies highlight bacterial pathogens also encode effectors with DUB activity (Sheedlo et al., 2015). These bacterial DUBs subvert the immune response by targeting the host’s ubiquitin system, thereby contributing to bacterial survival in host cells and enhancing bacterial pathogenicity (Pruneda et al., 2016; Qiu and Luo, 2017). For example, the *Salmonella* T3SS effector SseL represents the first reported bacterial DUB within the CE family. It exhibits the ability to hydrolyze both K48- and K63-linked polyubiquitin chains. Studies have demonstrated that SseL enhances *Salmonella* intracellular survival by suppressing the NF-κB signaling pathway and inflammatory responses, cleaving polyubiquitin chains on the *Salmonella*-containing vacuole (SCV) to inhibit autophagy, and reducing host cell cytotoxicity (Geng et al., 2019; Mesquita et al., 2012; Rytkönen et al., 2007). Similarly, *Chlamydia trachomatis* effectors ChlaDUB1 and ChlaDUB2 both possess DUB activity. Beyond deubiquitinating IκBα to inhibit NF-κB activation, ChlaDUBs also stabilize the anti-apoptotic protein Mcl-1, thereby modulating host cell apoptosis (Fischer et al., 2017). *Chlamydia pneumoniae* encodes an OTU-family DUB, ChlaOTU, which reduces polyubiquitin accumulation by binding to the autophagy receptor NDP52 (Fischer et al., 2017; Schubert et al., 2020). Furthermore, the *Legionella pneumophila* effector RavD specifically cleaves M1-linked linear ubiquitin chains on the *Legionella*-containing vacuole (LCV) within infected macrophages. This process attenuates NF-κB-mediated inflammatory signaling and promotes intracellular bacterial survival (Wan et al., 2019). However, it is predicted that *E. coli* also has a DUB called ElaD. Genome comparison results showed that ElaD is only present in all IPEC, but its function is unknown (Catic et al., 2007).

Here, we comprehensively describe the distribution of *elaD* in *E. coli*, and then connect *elaD* with the pathotypes, serotypes, phylogenetic groups and MLSTs of *E. coli*. We analyze the genetic and evolutionary relationships of ElaD in different *E. coli* strains, and elucidate the importance of ElaD in *E. coli* from the perspective of genomic analysis. Our findings provide insight into the distribution of ElaD and the basis for further research.

## Materials and methods

### Sequence information of *Escherichia coli* strains

The whole-genome of all *E. coli* strains were accessed from the database of the National Center for Biotechnology Information,^1^ from which 522 strains with known pathotypes were selected. Beside 522 strains from NCBI, we included 8 *E. coli* strains from different clinical tissue samples collected in our laboratory. These 530 strains were subjected to genome download or whole genome sequencing for bioinformatics analysis.

### Determination of *elaD* distribution by bioinformatics analysis

The complete genome sequences of 530 *E. coli* strains were used to create a local BLAST database, and the distribution rate of *elaD* in the genomes of the 530 strains was investigated by running BLAST. The gene was described as present if the result match three parameters simultaneously: identity>92%, query cover>90%, and E-value = 0.

### Analyses of pathotypes, serotypes, phylogenetic groups and MLSTs of *Escherichia coli* strains

To further elucidate the relationship between *elaD* distribution and *E. coli* pathotypes, serotypes, phylogenetic groups and MLSTs, each *E. coli* was determined: pathotypes were identified during the download of the *E. coli* whole-genome file by searching literatures and NCBI website details; serotypes were identified by comparison with EcOH database (Ingle et al., 2017) using ABRicate v.0.8;^2^ phylogenetic groups were interfered using the Clermon Typing method in silico (Beghain et al., 2018) and sequence typing (ST) was performed with the MLST scheme of Achtman (Jolley and Maiden, 2010) using mlst v.2.11 software.^3^

### Phylogenomic analysis of ElaD

Based on the ElaD amino acid sequences of all 353 *E. coli* strains, phylogeny was used for sequence analysis. After using ClustalW to match sequences under default parameters, a phylogenetic tree was constructed using the neighbor-joining method with 1,000 bootstrap values in MEGA 11 software. Phylogenetic tree was annotated and visualized using the Interactive Tree of Life (iTOLs) tool. Pathotypes, serotypes, phylogenetic groups and MLSTs of *E. coli* to which ElaD belongs were displayed alongside the phylogenetic tree.

### Statistical analysis

The chi-square test was used to identify differences in the prevalence of *elaD* gene of *E. coli* strains. All analyses were performed with SPSS version 16.

## Results

### Distribution of *elaD* genes in *Escherichia coli*

**Figure 1:**
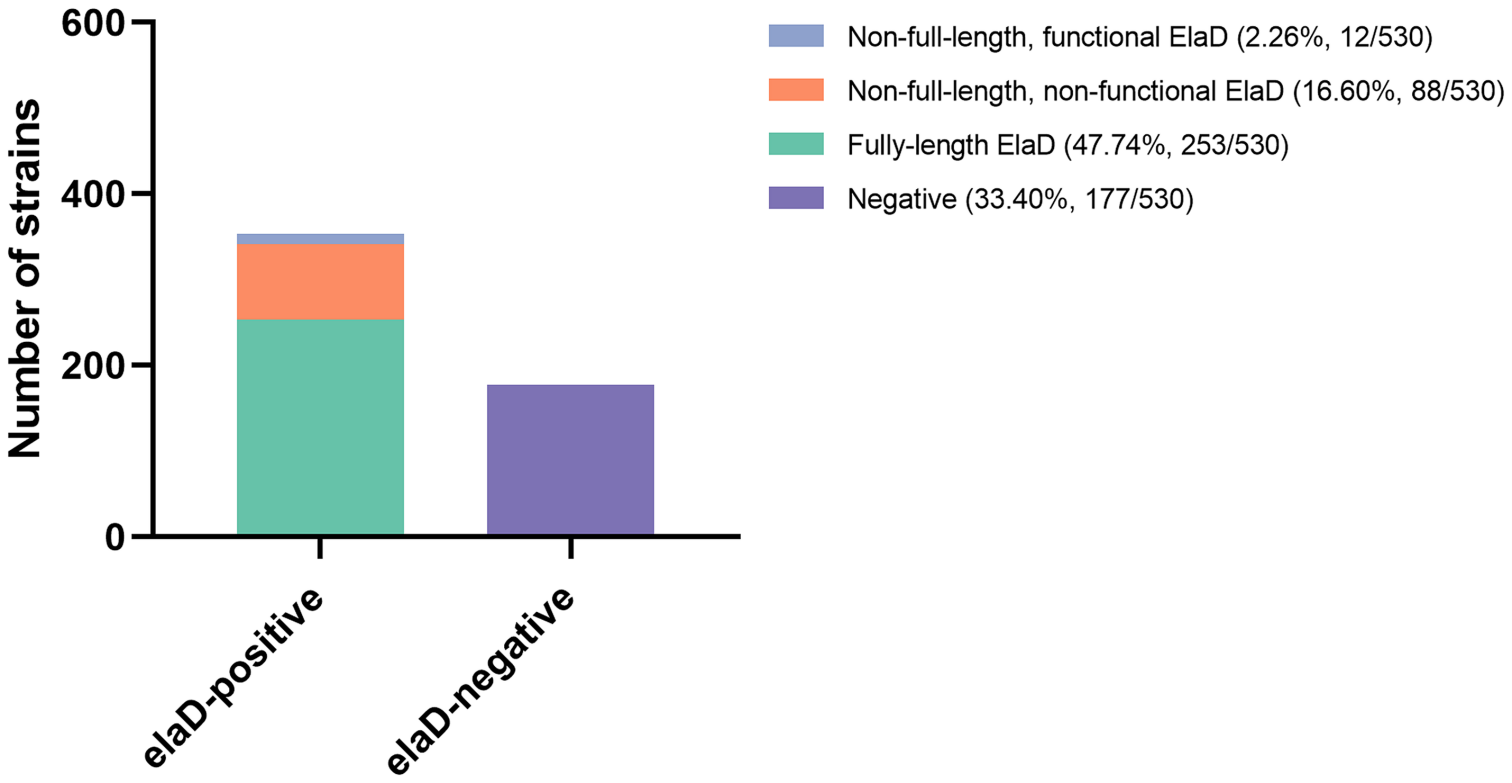
The distribution of *elaD* in *E. coli.* The distribution of the *elaD* gene in *E. coli* was confirmed using BLASTn, while the integrity of the protein encoded by the *elaD* gene was clarified by amino acid sequence analysis.

To examine the prevalence of the *elaD* sequence among *E. coli* strains, BLASTn was performed. The *elaD* gene was detected in 66.60% (353/530) of the *E. coli* strains. These *elaD* sequences shared >92% identity and were generally full length. Notably, further analysis of these *elaD* sequences revealed that 16.60% (88/530) of them matched the full-length reference gene, yet contained premature stop codons. These mutations prevent the translation of a full-length protein, suggesting that these variants are likely non-functional. Meanwhile, 2.26% (12/530) *elaD* sequences cannot encode full-length proteins but contain functionally relevant structural domains and could be considered functional. In addition, 33.40% (177/530) *E. coli* genomes did not match the *elaD* sequence at all (Figure 1). Therefore, ElaD is widely present in *E. coli* strains.

### The prevalence of ElaD in various *Escherichia coli* pathotypes

**Figure 2:**
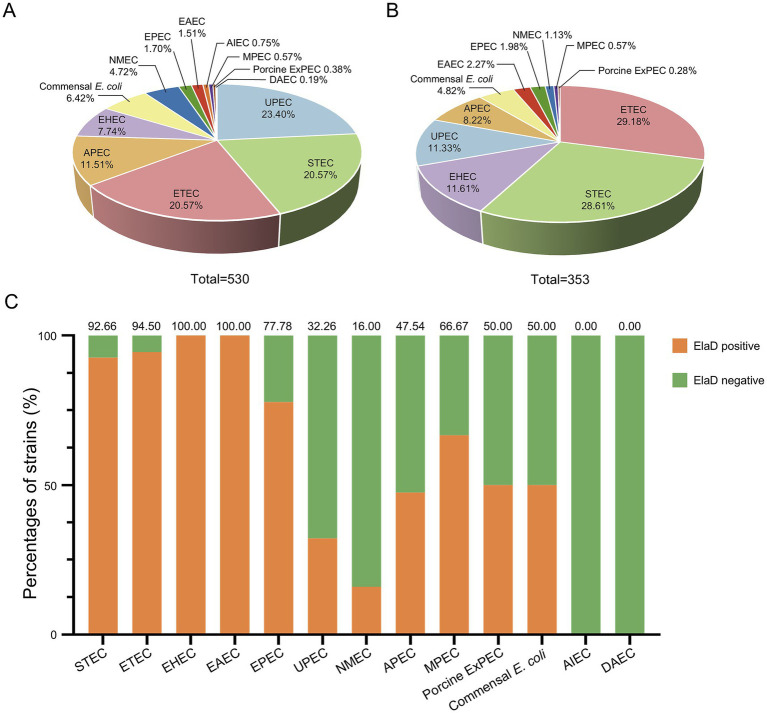
The prevalence of *elaD* in various pathotypes of *E. coli.* **(A)** The distribution of different pathotypes of all 530 *E. coli*. **(B)** The distribution of different pathotypes of 353 *elaD*-positive *E. coli* strains. **(C)** The *elaD* positivity for individual pathotypes in *elaD*-positive *E. coli* strains. The orange color represents the positive rate of *elaD* gene, while the green represents the negative rate.

In order to assign the *E. coli* strains to the different pathotypes, we summarized the details of all the strains (Table 1). As shown in Figure 2A, 13 pathotypes were identified among the 530 *E. coli* strains. 23.40% (124/530) of the strains were grouped as UPEC, 20.57% (109/530) as ETEC, 20.57% (109/530) as STEC, 11.51% (61/530) as APEC, 7.74% (41/530) as EHEC, 6.42% (34/530) as commensal *E. coli* and 4.72% (25/530) as NMEC; the remaining 27 strains (5.09%), including EPEC, EAEC, AIEC, MPEC, porcine ExPEC and DAEC, accounted for only a small percentage. Next, we determined the pathotype in the *elaD*-positive *E. coli* strains. Correspondingly, of the 353 *E. coli* strains positive for *elaD*, 29.18% (103/353) were ETEC, 28.61% (101/353) STEC, 11.61% (41/353) EHEC, 11.33% (40/353) UPEC, 8.22% (29/353) APEC, 4.82% (17/353) commensal *E. coli* and 6.23% others (Figure 2B).

**Table 1:** Distribution of the *elaD* gene in 530 *E. coli.*

Pathotypes	No. of strains	Total (*n* = 530)	
		No. of *elaD*-positive	No. of *elaD*-negative
**IPEC**	**281**	**260**	**21**
ETEC	109	103	6
STEC	109	101	8
EHEC	41	41	0
EAEC	8	8	0
EPEC	9	7	2
AIEC	4	0	4
DAEC	1	0	1
**ExPEC**	**215**	**76**	**139**
NMEC	25	4	21
UPEC	124	40	84
APEC	61	29	32
MPEC	3	2	1
Porcine ExPEC	2	1	1
**Commensal** *E. coli*	**34**	**17**	**17**
**Total**	**530**	**353**	**177**

However, the *elaD* was present in most of the STEC (92.66%, 101/109), ETEC (94.50%, 103/109), EHEC (100.00%, 41/41), EAEC (100.00%, 8/8) and EPEC (77.78%, 7/9). A feature clearly distinguishing ExPEC from IPEC was the lower occurrence of *elaD*, which was detected in only 66.67% of MPEC (2/3), 50.00% of porcine ExPEC (1/2), 32.26% of UPEC (40/124), 47.54% of APEC (29/61) and 16.00% of NMEC (4/25). Further categorical statistics have also confirmed this viewpoint, which is that *elaD* is distributed in 92.53% (260/281) of IPEC, but only exists in 35.35% (76/215) of ExPEC. The prevalence of *elaD* in IPEC strains was significantly higher than ExPEC (92.53% vs. 35.35%, p: <0.0001). But the gene was completely absent in AIEC and DAEC strains. It even presents in the 50.00% (17/34) commensal *E. coli*, which refers to harmless members of the normal intestinal bacterial microflora in humans and animals (Figure 2C).

### The prevalence of ElaD in various O serotypes

**Figure 3:**
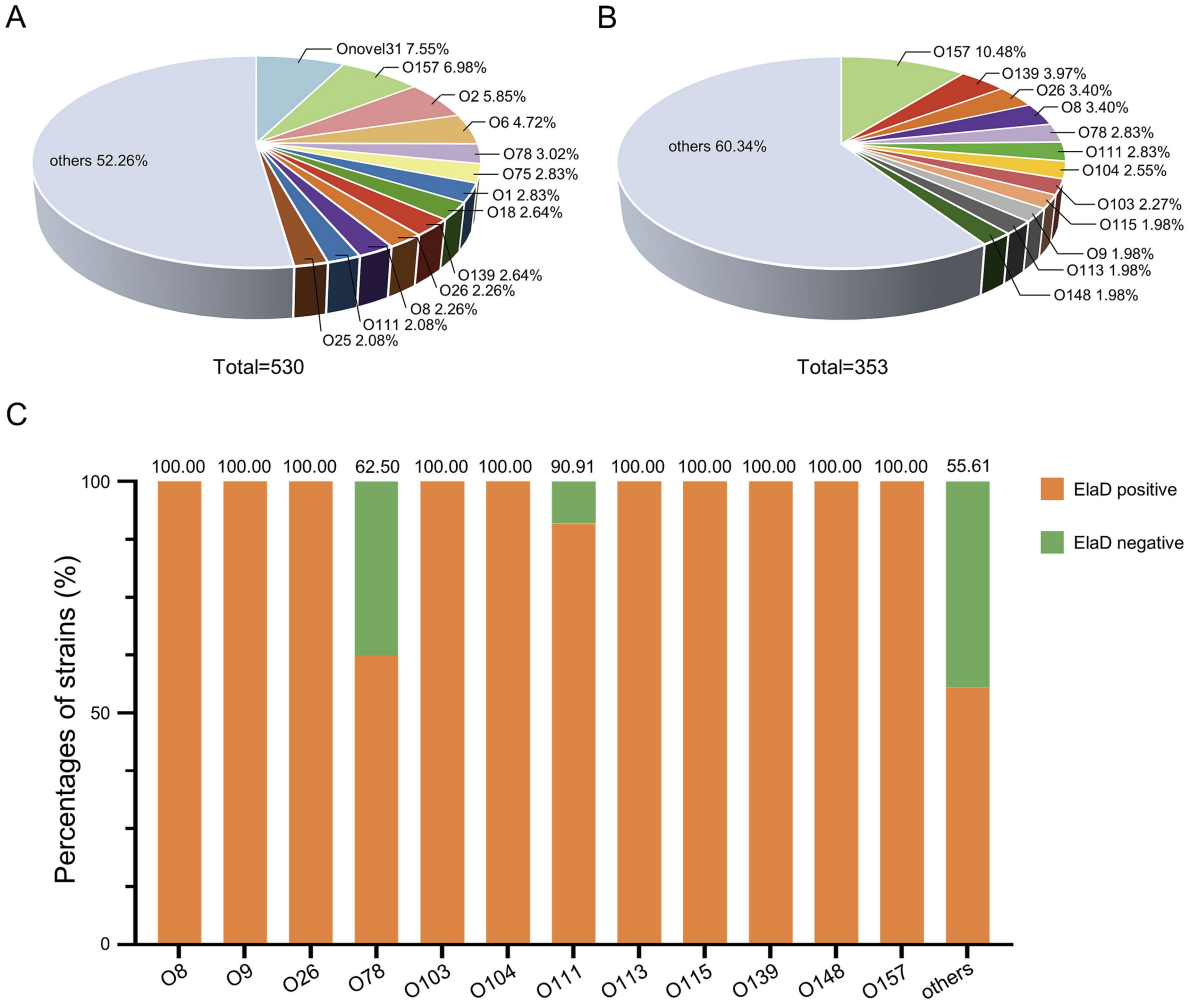
The prevalence of *elaD* in various O serotypes of *E. coli*. **(A)** The distribution of different O serotype of all 530 *E. coli*. **(B)** The distribution of different O serotype of 353 *elaD*-positive *E. coli* strains. **(C)** The *elaD* positivity for individual O serotype in *elaD*-positive *E. coli* strains. The orange color represents the positive rate of *elaD* gene, while the green represents the negative rate.

To compare the prevalence of *elaD* in various O serotypes, 530 strains were characterized using ABRicate v.0.8 software. Of these, 123 O serotypes were successfully identified, with Onovel31 (7.55%, 40/530) being the most prevalent one, followed by O157 (6.98%, 37/530), O2 (5.85%, 31/530), O6 (4.72%, 25/530), O78 (3.02%, 16/530), O1 (2.83%, 15/530), O75 (2.83%, 15/530), O139 (2.64%, 14/530), O18 (2.64%, 14/530), O26 (2.26%, 12/530), O8 (2.26%, 12/530), O111 (2.08%, 11/530) and O25 (2.08%, 11/530), while 52.26% were distributed among an additional 110 O-types (Figure 3A). *e*laD gene is found in the O157 (10.48%, 37/353), O139 (3.97%, 14/353), O26 (3.40%, 12/353), O8 (3.40%, 12/353), O78 (2.83%, 10/353), O111 (2.83%, 10/353), O104 (2.55%, 9/353), O103 (2.27%, 8/353), O115 (1.98%, 7/353), O9 (1.98%, 7/353), O113 (1.98%, 7/353), O148 (1.98%, 7/353) and strains from the other 111 O-types (Figure 3B).

The distribution of *elaD* genes for each O serotype strains are shown in Figure 3C. The *elaD* positivity rate was 100.00% across all 89 O serotypes (including O8, O9, O26, O103, O104, O113, O115, O139, O148 and O157). However, *elaD* was rarely present in all Onovel31, O75 and O18 serotype strains detected. These results suggest that there is a correlation between *E. coli elaD* and O serotypes, especially the O157, O139 and O26 serotypes.

### The prevalence of ElaD in various phylogenetic groups

**Figure 4:**
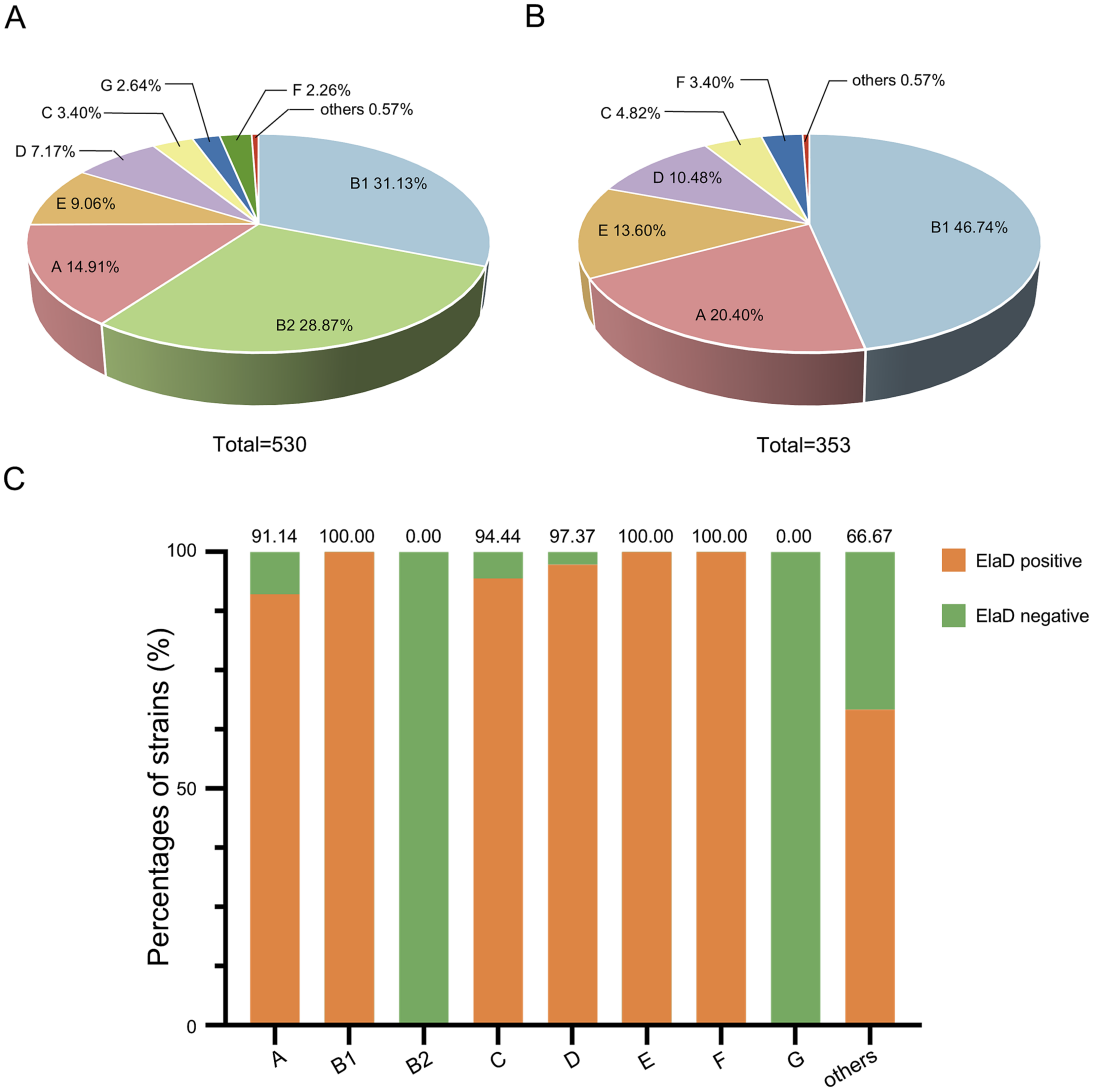
The prevalence of *elaD* in various phylogenetic groups of *E. coli*. **(A)** The distribution of different phylogenetic group of all 530 *E. coli*. **(B)** The distribution of different phylogenetic group of 353 *elaD*-positive *E. coli* strains. **(C)** The *elaD* positivity for individual phylogenetic group in *elaD*-positive *E. coli* strains. The orange color represents the positive rate of *elaD* gene, while the green represents the negative rate.

The distribution of the 530 collected *E. coli* strains among the phylogenetic groups was determined using the Clermon Typing method and is shown in Figure 4A. Most of the strains could be categorized into groups B1 (31.13%, 165/530) and B2 (28.87%, 153/530), while other strains belonged to groups A (14.91%, 79/530), E (9.06%, 48/530), D (7.17%, 38/530), C (3.40%, 18/530), G (2.64%, 14/530) and F (2.26%, 12/530). 3 strains (0.57%) were not assigned to any group. A comparison of the distribution of the phylogenetic groups among *elaD*-positive *E. coli* revealed that 46.74% (165/353), 20.40% (72/353), 13.60% (48/353), 10.48% (38/353), 4.82% (17/353) and 3.40% (12/353) of the *elaD*-positive strains were assigned to groups B1, A, E, D, C and F, respectively (Figure 4B).

The analyses showed a clear correlation between the distribution of the *elaD* gene in *E. coli* and the phylogenetic group. The *elaD* gene was found in all B1, E and F examined in our *E. coli* database. Notably, our results showed that *elaD* was found in none of the groups B2 and G. In addition, *elaD* gene was present in more than 90% of the A, C and D *E. coli* (Figure 4C).

### The prevalence of ElaD and association of MLST

**Figure 5:**
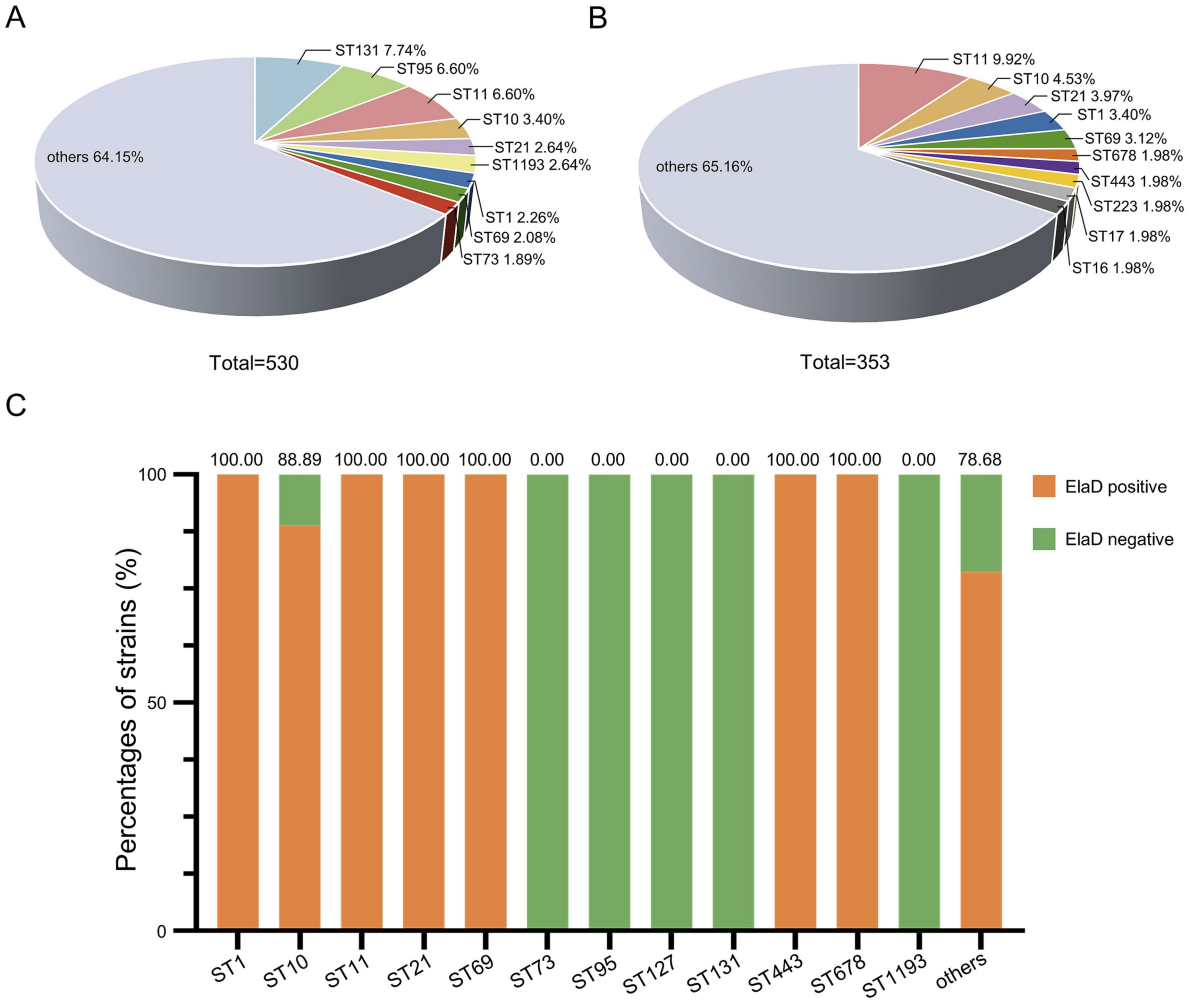
The prevalence of *elaD* in various MLSTs of *E. coli*. **(A)** The distribution of different MLSTs of all 530 *E. coli*. **(B)** The distribution of different MLSTs of 353 *elaD*-positive *E. coli* strains. **(C)** The *elaD* positivity for individual MLSTs in *elaD*-positive *E. coli* strains. The orange color represents the positive rate of *elaD* gene, while the green represents the negative rate.

The conventional seven-gene MLST analysis revealed that the strains were relatively diverse with a total of 174 STs in the 530 *E. coli* strains, among which ST131 comprised 7.74% (41/530) of the strains in this study, followed by ST95 (6.60%, 35/530), ST11 (6.60%, 35/530), ST10 (3.40%, 18/530), ST21 (2.64%, 14/530), ST1193 (2.64%, 14/530), ST1 (2.26%, 12/530), ST69 (2.08%, 11/530) and ST73 (1.89%, 10/530), while 64.15% were distributed among an additional 165 STs (Figure 5A). In contrast, the distribution of STs of 353 *elaD*-positive *E. coli* strains was 9.92% ST11 (35/353), 4.53% ST10 (18/353), 3.97% ST21 (14/353), 3.40% ST1 (12/353), 3.12% ST69 (11/353), 1.98% ST678 (7/353), 1.98% ST443 (7/353), 1.98% ST223 (7/353), 1.98% ST17 (7/353), 1.98% ST16 (7/353) and 65.16% others, which are almost the same STs mentioned earlier to predominate among 530 *E. coli* strains (Figure 5B).

The *elaD* gene was present in all tested strains of ST1, ST11, ST21, ST69, ST443 and ST678 (including most of the other STs identified), demonstrating a strong association between *elaD* and *E. coli* MLST. Surprisingly, ST73, ST95, ST127, ST131 and ST1193 *E. coli* appear to completely lack the *elaD* gene (Figure 5C).

### Phylogenetic analysis of the ElaD of *Escherichia coli*

To further elucidate the distribution of the *elaD* in *E. coli*, we constructed a phylogenetic tree using the ElaD amino acid sequence of 353 strains. The pathotypes, serotypes, phylogenetic groups and MLSTs of the 353 strains were presented along with the phylogenetic tree and displayed with distinguishable colors and stripes.

**Figure 6:**
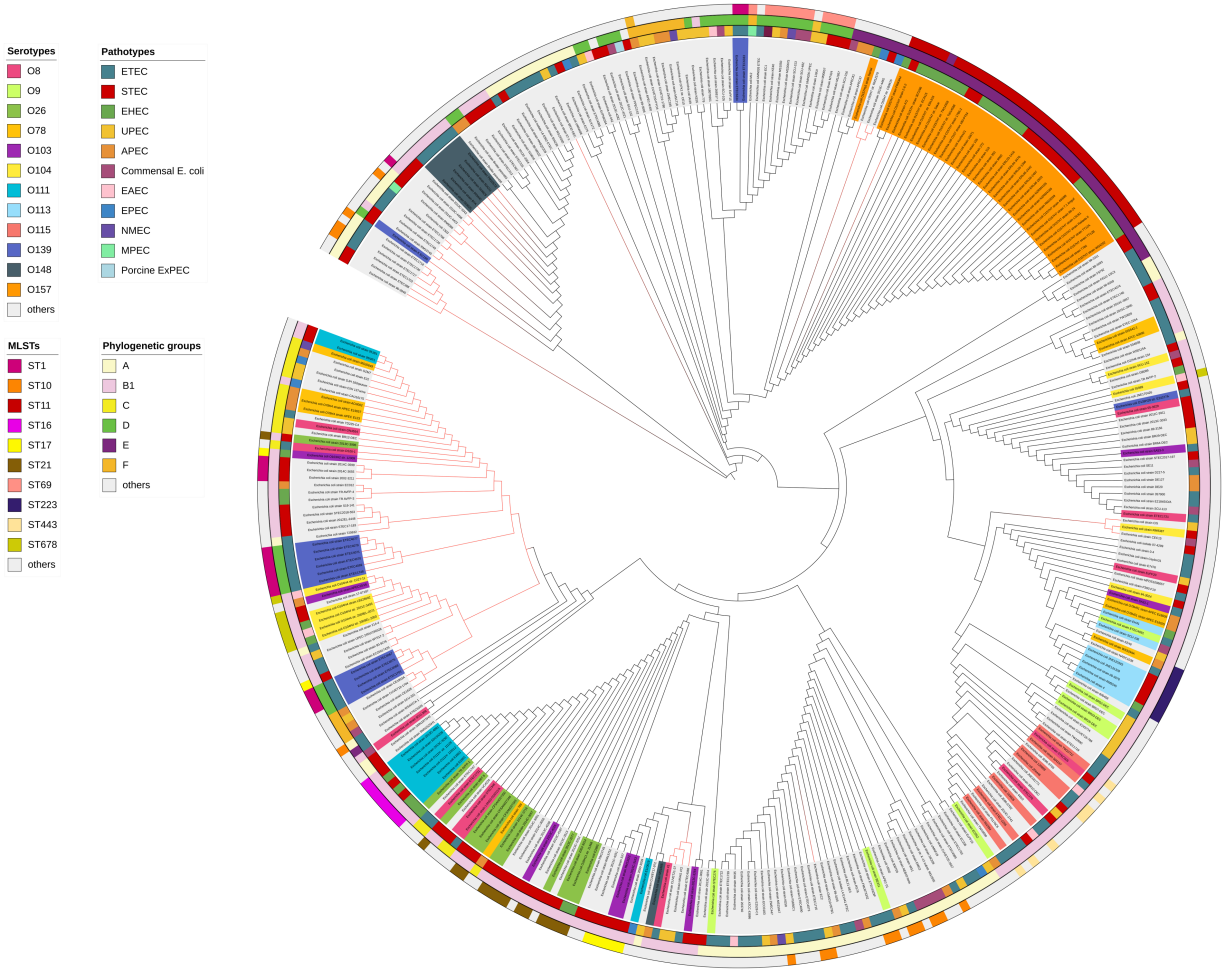
ElaD amino acid sequences based phylogenetic tree of 353 *E. coli* strains. The color range of the ring shows the names of the individual strains and their O serotypes. Then, the color strips from the innermost to the outermost represent the pathotypes, phylogenetic groups and MLSTs of *E. coli* strains, respectively. Diverse type is marked in different colors. In addition, the red branches represent the non-full-length amino acid sequence translated by the gene.

The ElaD amino acid sequences of 353 strains were extensively distributed across the phylogenetic tree, of which 53 and 22 ElaD showed premature translation termination and were clustered in two regions, respectively. This phenomenon suggested that the *elaD* gene may have mutated during evolution leading to premature termination of translation. However, the remaining 13 ElaD proteins with premature translation termination were scattered in other clades. There were no major clusters of STs on the tree, except for ST11. Likewise, overlaying information on the pathotype from which these *E. coli* strains were derived revealed no clear relevance between placement within the phylogeny and bacterial pathotype. The O antigens, such as O157, O148, O111 and O139, corresponded with the phylogenomic results to a certain extent, however, other O antigens are interspersed in the tree. Furthermore, by analyzing the correlation between the bacterial phylogenetic group and the ElaD, we found that the ElaD from the same phylogenetic group are generally on the same clade, such as A, B1 and E (Figure 6).

## Discussion

Pathogenic *E. coli* can cause a wide range of diseases, from gastroenteritis and diarrhea to extraintestinal infections, threatening worldwide human health and the development of the livestock farming (Croxen and Finlay, 2010). During infection, the secretion system can deliver effectors to host cells that interfere with specific cellular and host immune responses (Green and Mecsas, 2016). A number of bacterial pathogens have been shown to encode and deliver effectors with DUB activity, which promote bacterial survival and enhance pathogenicity by targeting and disrupting the host ubiquitination system. Recently, ElaD has been identified as a DUB in *E. coli*. However, the distribution and evolution of this gene in *E. coli* remains unknown.

This study showed that 66.60% of the 530 *E. coli* strains analyzed contained the *elaD* gene. Meanwhile, the detection rate of *elaD* exceeded 70% in all five collected IPEC pathotypes—namely EHEC, STEC, ETEC, EAEC and EPEC. These pathotypes of *E. coli* can cause acute and persistent diarrhea in children, adults and other mammals (calves, piglets, etc.), and in severe cases, lead to hemorrhagic colitis or lethal hemolytic uremic syndrome, which is a health hazard (Bai et al., 2016; Kolenda et al., 2015; Kolodziejek et al., 2022). IPEC primarily relies on intestinal adhesion, colonization and the modulation of inflammatory responses to promote bacterial survival and replication, which ultimately contribute to disease development (Jesser and Levy, 2020). This process relates to various adhesins, toxins and effectors. The type III secretion system (T3SS) or *E. coli* type III secretion system 2 (ETT2) has been identified in various IPEC, which can directly deliver effectors into host cells (Gaytán et al., 2016; Wang et al., 2024). Furthermore, previous studies have compared ElaD with deubiquitinases from other bacteria in terms of evolution, structure, and function. Phylogenetic analyses indicate that *E. coli* ElaD within the same clade as *Legionella pneumophila* effector and *Salmonella Typhimurium* SseL, showing high sequence homology. It has been demonstrated that ElaD possesses deubiquitinating enzyme activity (Catic et al., 2007). Given that SseL has been confirmed as a T3SS effector that inhibit host inflammatory responses via its deubiquitination activity, we hypothesize that the high detection rate of *elaD* in IPEC might also indicate that the presence of *elaD* contributes to the infection process of IPEC.

A previous study showed that *elaD* was only present in commensal *E. coli* K12 and all IPEC in 16 sequenced *E. coli* strains. However, our results found that ExPEC also possessed *elaD* but its detection rate was relatively low, about 35.35%. In addition, *elaD* was also present in other commensal *E. coli*. The wide distribution of *elaD* in different pathogenic *E. coli* demonstrates its significance.

Our results revealed a high distribution of *elaD* among the O157, O139, O26, O8, O111 and O78 serotypes. These six serotypes are known to be closely associated with human and animal disease, with O157 considered to be the major serotype responsible for serious foodborne disease caused by STEC and EHEC infections worldwide, while the non-O157 serotypes, including O26 and O111, are equally important in causing outbreaks of intestinal infections (Mathusa et al., 2010; Wang et al., 2013). ETEC serotypes O8 and O139 are considered to be the main diarrheal pathogens affecting pigs below four weeks of age (Wyrsch et al., 2015). Meanwhile, O78 is the predominant serotype in APEC strains. The detection rate of the *elaD* gene was 100% for all 4 O serotypes, except for O111 (90.91%) and O78 (62.50%). The correlation between the distribution of *elaD* and bacterial serotypes also demonstrates the importance of ElaD.

A close relationship between phylogenetic group and virulence factors (VFs) of the pathogens was reported. EHEC, ETEC and STEC/EIEC and their specific VFs were found only in A, B1, C or E groups. In contrast, ExPEC strains and their VFs preferentially belonged to B2 and D group (Escobar-Páramo et al., 2004). Our study also demonstrated the relationship between the distribution of the *elaD* gene and the phylogenetic group of the pathogens. The positivity rates of *elaD* in *E. coli* was more than 90.00% in A, B1, C, D, E and F groups. Notably, in all B2 and G groups *E. coli* detected, the *elaD* gene was absent. It further suggests the notion that the presence of the *elaD* gene on IPEC or ExPEC is correlated with its pathogenic potential.

Furthermore, the MLST results offer a crucial reference for our analysis. ST11 represents a lineage of *E. coli* that is primarily associated with the O157: H7 serotype (Pugh et al., 2023). Our findings revealed that the *elaD* gene was present in all examined ST11 *E. coli* strains, all of which belonged to the O157 serotype and the E group. This is also evidenced in the phylogenetic tree.

ST131, initially recognized for its association with the carriage of extended-spectrum *β*-lactamase genes, has emerged as the predominant ST among global ExPEC isolates, belonging to phylogenetic group B2 (Nicolas-Chanoine et al., 2014). Notably, the *elaD* gene was completely absent in all examined ST131 strains, which corresponds to a 100% *elaD* negativity rate within the B2 group. Likewise, ST95 strains belonging to phylogroup B2 do not carry the *elaD* gene (Xia et al., 2022). During the long-term evolution, *E. coli* genome exhibits frequent alterations by increased rates of homologous recombination or horizontal gene transfer (Mageiros et al., 2021). These genes stably gained or lost, contribute to the fitness of the group B2 strains (Touchon et al., 2009). Therefore, the absence of *elaD* in the B2 group ExPEC may reflect the loss of adaptive genes, which may be caused by environmental pressure or niche specialization. Besides, ExPEC strains usually translocate from the gut to colonization sites, evade the host’s defense system (such as complement and phagocytosis) and establish persistent infection. ExPEC can even spread in bloodstream and cause fatal multisystemic infection (Biran et al., 2021). During this process, ExPEC employs various virulence factors, including adhesins, invasins, iron uptake factors, protectines, and toxins, which can cooperate and contribute to the pathogenic potential (Sora et al., 2021). Therefore, we hypothesize that the pathogenic process of these B2 group ExPEC may not primarily depend on ElaD. Further research is essential to comprehensively investigate the distribution and function of ElaD across *E. coli*.

In this study, *E. coli* strains showed a high prevalence of the *elaD* gene. Its presence correlating significantly with specific bacterial pathotypes, serotypes, phylogenetic groups, and MLSTs. Our results tentatively suggest that ElaD is strongly associated with bacterial pathogenicity, especially IPEC, but the specific regulatory mechanism by which the ElaD will act as a DUB has not yet been clarified. Further studies are still needed to evaluate the role of ElaD in the pathogenic *E. coli* to help us resolve the mechanism of *E. coli* infections and provide a reference for the prevention of *E. coli* disease and potential human and animal infections.

## Data Availability

The datasets presented in this study can be found in online repositories. The names of the repository/repositories and accession number(s) can be found in the article/Supplementary material.

## Footnotes

^1^ https://www.ncbi.nlm.nih.gov

^2^ https://github.com/tseemann/abricate

^3^ https://github.com/tseemann/mlst
